# *p*-Glycoprotein ABCB5 and YB-1 expression plays a role in increased heterogeneity of breast cancer cells: correlations with cell fusion and doxorubicin resistance

**DOI:** 10.1186/1471-2407-10-388

**Published:** 2010-07-22

**Authors:** Ji Yeon Yang, Seon-Ah Ha, Yun-Sik Yang, Jin Woo Kim

**Affiliations:** 1 Molecular Genetic Laboratory, College of Medicine, The Catholic University of Korea, Seoul 137-040, Republic of Korea; 2 Genome Research Center for Immune Disorders, School of Medicine, Wonkwang University, Iksan, Republic of Korea; 3 Department of Obstetrics and Gynecology, College of Medicine, The Catholic University of Korea, Seoul 137-040, Republic of Korea

## Abstract

**Background:**

Cancer cells recurrently develop into acquired resistance to the administered drugs. The iatrogenic mechanisms of induced chemotherapy-resistance remain elusive and the degree of drug resistance did not exclusively correlate with reductions of drug accumulation, suggesting that drug resistance may involve additional mechanisms. Our aim is to define the potential targets, that makes drug-sensitive MCF-7 breast cancer cells turn to drug-resistant, for the anti-cancer drug development against drug resistant breast cancer cells.

**Methods:**

Doxorubicin resistant human breast MCF-7 clones were generated. The doxorubicin-induced cell fusion events were examined. Heterokaryons were identified and sorted by FACS. In the development of doxorubicin resistance, cell-fusion associated genes, from the previous results of microarray, were verified using dot blot array and quantitative RT-PCR. The doxorubicin-induced expression patterns of pro-survival and pro-apoptotic genes were validated.

**Results:**

YB-1 and ABCB5 were up regulated in the doxorubicin treated MCF-7 cells that resulted in certain degree of genomic instability that accompanied by the drug resistance phenotype. Cell fusion increased diversity within the cell population and doxorubicin resistant MCF-7 cells emerged probably through clonal selection. Most of the drug resistant hybrid cells were anchorage independent. But some of the anchorage dependent MCF-7 cells exhibited several unique morphological appearances suggesting minor population of the fused cells maybe de-differentiated and have progenitor cell like characteristics.

**Conclusion:**

Our work provides valuable insight into the drug induced cell fusion event and outcome, and suggests YB-1, GST, ABCB5 and ERK3 could be potential targets for the anti-cancer drug development against drug resistant breast cancer cells. Especially, the ERK-3 serine/threonine kinase is specifically up-regulated in the resistant cells and known to be susceptible to synthetic antagonists.

## Background

The prognosis of breast cancer patients is closely associated with the response of the tumor cells to chemotherapy. Doxorubicin is one of the primary chemotherapeutic agents used for the treatment of breast cancer [1,2]. Various tumors initially respond to administered drugs, however, once the cancer cells could gain resistance during anticancer drug treatment, there are only a few treatment options [3,4]. Resistance to chemotherapy is believed to cause treatment failure in over 90% of the patients with metastatic cancer [5,6]. Multidrug resistant cancer cells are thought to be derived from emerging clones within primary tumors [7] and all multidrug resistances were found to be closely related with additional changes to the already altered copy number profile of the breast tumors [8,9]. Up to now, the iatrogenic mechanisms of induced chemotherapy-resistance and the question of whether cells with drug resistance potential are frequent or atypical within human cancers remain mainly elusive.

Interestingly, cancer cells were known to fuse with many cell types *in vivo*, including stromal cells and endothelial cells [10,11]. Cell fusion was reported to account for the plasticity of adult stem cells *in vivo *[12], while an accidental heterotypic cell fusion was noted to build tetraploid cells that may lead to initiation and progression of neoplastic tumor through genomic instability [13]. However, the mechanism of action of doxorubicin appears to be complex and unclear, moreover, doxorubicin was known to interact directly with tumor cell membrane proteins.

Recently, Y-box binding protein-1 (YB-1) was reported as a stronger predictor, of all breast tumor subtypes specific survival, than estrogen receptor or HER-2 [14]. The locus of YB-1 gene is on 1p34 and 80% of primary breast tumors show increased copy numbers of chromosome 1 [15]. When cells are under the cytotoxic stimuli of anti-cancer drugs, the stress-responsive protein YB-1 is known to be translocated to the nuclei [16], YB-1 functions as a transcription factor for the expression of the well known membrane efflux pump, the multiple drug resistance gene I (MDR1/ABCB1) that allows anti-cancer drug resistance [17].

Recently, however, the resistance mechanisms are revealed as multifactorial and no single protein expression is solely responsible for acquired multiple drug resistance [18-21]. Amino acid sequence of *p*-Glycoprotein ABCB5 was reported highly homologous to both of the known human P-gp isoforms ABCB1 (MDR1) and ABCB4 (MDR3), and new evidence has suggested that ABCB5 could mediate cell to cell fusion [22]. On the basis of this rationale and with our preliminary results from microarray analysis on cell fusion (data not shown), we postulated that cell fusion could be an essential prerequisite event prior to diversified MCF-7 subpopulations emerging.

Therefore, the goals of the present study were, firstly, to investigate whether the overexpression of YB-1 could induce cell fusion on doxorubicin treatment, secondly, to scrutinize whether this cell fusion event could increase genetically diversified cell population, and finally, to define the potential targets of pro-survival gene products and pro-apoptotic ones (that makes drug-sensitive MCF-7 breast cancer cells turn to drug-resistance).

## Methods

### Cell culture and drug selection

**Figure 1:**
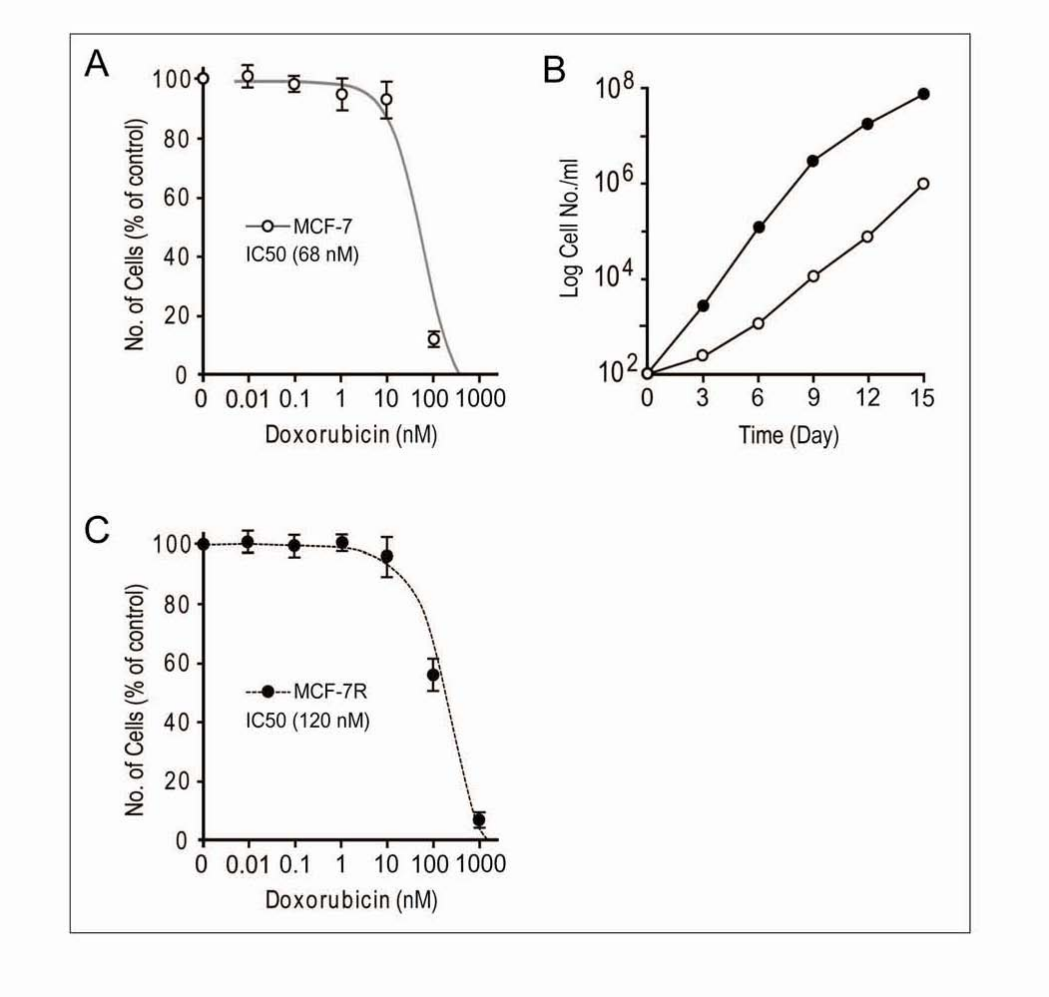
**Effect of different concentration of doxorubicin treatments on the survival of MCF-7 breast cancer cell lines**. A and C: Viability of MCF-7 cells in the presence of a series of concentrations of doxorubicin (0.01-1000 nM) for two weeks, the percent viability was measured following the standard protocols (non-radioactive cell proliferation assay kit, Promega). B: Control MCF-7 cells; black circlet (●), doxorubicin-treated cells; white circlet (**○**). Time dependence effect response of doxorubicin (10 nM) on cellular growth rate of MCF-7 cells was assayed by comparing the each value with control. For the counting of colony number, cells were stained with 1% methylene blue for 20 m and washed with water. Each data point is the average of at least three independent experiments performed.

Human breast MCF-7 carcinoma cells were grown in Dulbecco's modified Eagle's medium (GIBCO) supplemented with 10% (v/v) fetal bovine serum, 1% penicillin/streptomycin (Gibco) in standard culture conditions (95% air-5% CO_2_, 37°C). The cells were inspected on a daily basis with inverted microscope and documented with digital camera (Leica, DM IRB/DC300). 100 μl MCF-7 cells (2 × 10^4 ^cells/ml) were distributed into each well of 96 well plates (Becton Dickinson, USA) and allowed to adhere 18 hours and further incubated for two weeks with increasing concentrations (0.01--1000 nM) of doxorubicin hydrochloride (Duchefa), to a final volume of 200 μl per well, then MTS reagent (The CellTiter 96^® ^AQ_ueous _Non-Radioactive Cell Proliferation Assay kit, Promega) was added into each well and incubated for 2 h before reading at a wavelength of 490 nm. A 50% growth inhibition (IC_50_) values for doxorubicin were calculated from dose-response curves obtained from the three independent experiments (Figure 1A and 1C). We generated doxorubicin resistant MCF-7 cell clones following the single-step selection procedure with the optimized low dose-effect of doxorubicin (10-20 nM). Then, the resistant clones were maintained in the medium with or without doxorubicin. The resistant phenotype of isolated clones, after the single-step selection, was re-examined 24 weeks later and confirmed to be stable. Time dependent effect of doxorubicin, on cellular growth rate of MCF-7 cells, was examined by incubating ~1000 cells with 10 nM doxorubicin for 15 days (with 3 day intervals) and the ratios of viable cell percent were calculated by comparing the control with the values of each days. After trypsinization, cells were collected by centrifugation, then resuspended in PBS buffer. The cell suspension was mixed with an equal volume of trypan blue solution (0.4%). Each sample was counted in triplicates with hemacytometer. Stained (dead) and unstained (viable) cells were counted with an inverted microscope.

### Creation of GFP-YB-1 and DsRed-YB-1 expressing MCF-7 cell lines

The human YB-1 coding sequence (containing 1.4 kb YB-1) was amplified from pCMV-SPORT6/YB-1 (a generous gift from Yun-Sik Yang, Genome Research Center for Immune Disorders, Wonkwang University) and inserted into *EcoRV */*Xho*1 of multiple cloning site (MCS) of a pcDNA3-EGFP vector (6.2 kb) and into *EcoR*1 and *Apa*1 of a pDsRed2-N1 vector (4.7 kb, BD Clontech). The vectors, pcDNA3-EGFP/YB-1 and pDsRed2-N1/YB-1 (having the initiator ATG codon of YB-1), direct expressions of fusion proteins, GFP-YB-1 and DsRed-YB-1, respectively under CMV promoter in MCF-7 cells. For that purpose, 1 × 10^5 ^MCF-7 cells were grown on a 6-well plate for 24 h and the expression constructs were transfected into doxorubicin sensitive MCF-7 cells according to the manufacturer's instructions (FuGENE 6, Roche). 48 h after transfection, G418 (500 μg/ml, Duchefa) was added for the selection of the stably transfected cells and continued for 2 weeks. The efficiency of the transfection was evaluated 22 hours after transfection by observing the green GFP and red DsRed fluorescence in the transfected cells using an Axiovert 200 fluorescent microscope (Zeiss), the images captured using a digital camera (AxioCam MRm) and visualized and documented using the AxioVision Rel 4.6 software.

### Dot blot array assay analysis

Based on the knowledge gained from the microarray assay (data not shown) on cell fusion, custom made dot blot arrays were employed to verify whether the up-regulated genes on fused cell hybrids are biologically relevant in the development of doxorubicin resistance. The methods of array assay were adopted from the Atlas Select Human Tumor Array (Clontech, PT1117-1, PT3399-1) with modifications. The gene specific primers were designed with MacVector software (International Biotechnologies) employing a sequence data base. The primers were as follows: YB-1 forward, 5'-tcgcagtgtaggagatggagagact-3'; YB-1 reverse, 5'-cggtaccgacgttgaggtggct-3'; c-Kit forward, 5'-ggcgcgagggaggggaggcgagga-3'; c-Kit reverse, 5'-aagtgcagcgagcgcggcaaagcc-3'; MAPT forward, 5'-cccaacactcctcagaacgaacttatcc-3'; MAPT reverse, 5'-gccatcctggttcaaagttca-3'; GST forward, 5'-ttggccatgacgcgggttgt-3'; GST reverse, 5'-agttgggcacagaaacaaatcttgga-3'; GM-CSF2RA forward, 5'-agatctgacagcctgaaccctcct-3'; GM-CSF2RA reverse, 5'-gtgtcgcggaggcggagatg-3'; 15-LOX-1 forward, 5'-ggctcgggaccaggtttgcc-3'; 15-LOX-1 reverse, 5'-gtggcttgggtgatgggggc-3'; CypB forward, 5'-gagggcatggaggtggtgcg-3'; CypB reverse, 5'-gccagtgcagctcagagccc-3'; LCN2 forward, 5'-tcccagccccacctctgagc-3'; LCN2 reverse, 5'-atggtgttcgggctggtgcg-3'; GRP78 forward, 5'-aagcgccgcggcctgtattt-3'; GRP78 reverse, 5'-acgccgacgcaggagtaggt-3'; RSK1 forward, 5'-cgtccactgggaccaactgcc-3'; RSK1 reverse, 5'-agcagcagcaggaacagcagc-3'; COX2 forward, 5'-ccaggcgacctgcgactcct-3'; COX2 reverse, 5'- accgtagtatacccccggtcgtg-3'; CRABP2 forward, 5'-tgctcaaagtgctgggggtga-3'; CRABP2 reverse; 5'-gcgcacggtggtggaggttt-3'; Gapdh forward, 5'-ccaaacgggtcatcatctc-3'; Gapdh reverse, 5'-tggatgcagggatgatgtt-3'; β-actin forward, 5'-cctggcacccagcacaat-3' and β-actin reverse, 5'-gccgatccacacggagtact-3'. For the cDNA amplification of target genes, template was generated from polyA^+^-RNA or genomic DNA of MCF-7 cells that were serially diluted to give an optimal concentration for PCR. The subsequent PCR products were evaluated by gel electrophoresis on 1~2% agarose gels and confirmed a single PCR product of the predicted size. Each 100 pmol of 14 cDNA products were spotted onto nylon membranes (Hybond-N^+^, Amersham) with QArray systems (Genetix) in duplicates to eliminate potential non-specific single spot hybridization. On the other hand, total RNA was recovered with TRIzol reagent (Invitrogen) followed by isopropanol precipitation and ethanol washing from the each of doxorubicin sensitive MCF-7 cells (5 × 10^6^), after 0, 2, 4, 6, 12, 24 and 48 h of incubation with 20 nM doxorubicin. Second purification was done by RNeasy Protect Mini kit (Qiagen), following the manufacturer's protocol. For cDNA labeling, total RNA was converted into α-32P-labeled first strand cDNA with gene-specific primer cocktails. After hybridization, the membrane was quickly washed, then exposed to a phosphor imaging plate (Molecular Dynamics) for three to five days. ImageQuant version 1.2 (Molecular Dynamics) was used for visualization and spot finding, spot quantification. The house keeping genes (Actin, GAPDH) were employed for normalization. For background subtraction and normalization, the Excel (Microsoft) program was used. Spots below threshold intensity were eliminated and the expression ratio data were median-centered.

### Microarray Assays

Total RNA (150 μg) was extracted from each of the non-fused and fused MCF-7 cells (R2, FACS sorted) and assayed with a Human Sentrix-6 V3 BeadChip that containing 48,803 human genes (Illumina, GenoCheck, Korea).

### Flow cytometry

In order to analyse the cell to cell fusion of the stably strasfected cells (with pcDNA3-EGFP/YB-1 and pDsRed2-N1/YB-1), the two transfectants were mixed (1 to 1 ratio) and co-cultured with or without 10 nmol/L doxorubicin for 6 days and 1 × 10^7^/ml cells were resuspended in (1×) PBS for immediate analysis or in (1×) PBS containing 5% FBS for cell sorting. Fluorescence was detected using a MoFlow flow cytometer/cell sorter (Beckman Coulter) and analyzed using Summit 5.1 software. Dead cells were gated out first and hetertypically fused cells gated and sorted based on merged fluorescence of GFP and DsRed.

### Western blot analysis

The same numbers of MCF-7 cells (5 × 10^7^) were lysed using 150 μl RIPA buffer with freshly added protease inhibitors, then transferred to an Eppendorf microcentrifuge tube and mixed by inversion for 30 m at 4°C and centrifuged at 14000 rpm for 30 m. The supernatant was transferred to a new Eppendorf tube, the protein concentration was determined by the Bradford method. The protein extracts were electrophoresed by 12% SDS-PAGE, then electrically transferred onto nitrocellulose filters and probed with the following primary antibodies (specific to the proteins that may associated with acquired drug resistance): YB-1 (dilution factor 1:200, Cell Signalling), c-Kit (1:1000, Cell Signalling); ERK1/2 (1: 1000, Cell Signalling); ERK3 (1: 500, Novus Biologicals); FAS (1: 500, Santa Cruz Biotechnology); MAPT (1: 1500, Proteintech Group); and MDR1 (1: 500, Santa Cruz Biotechnology), ABCB5 (1: 2000, ProSci); PARP-1 (1: 2000, Trevigen); β-Actin (1:5000, Sigma) was used as a loading control. The membranes were rinsed with Tris-buffered saline, treated with horseradish peroxidase-conjugated secondary antibody (Jackson ImmunoResearch Laboratories) was applied for 1 h at 1:2000 dilution. After four washes in PBST, blots were treated with ECL-plus (Amersham Pharmacia) according to the manufacture's specification, and exposed to Biomax ML film (Kodak) to detect the protein bands. The intensity of the band derived from each sample was measured by AlphaImager^® ^HP densitometer.

## Results

### Increased genomic instability of MCF-7 cells during single-step low dose doxorubicin selection

Figure 1A and 1C shows percentage vitality of MCF-7 cells. The dose-effect of doxorubicin (0.01, 0.1, 1, 10 and 1000 nM) on cellular growth rate of MCF-7 was observed over the period of two weeks. Our proximal 50% growth inhibition (IC_50_) of doxorubicin for parental MCF-7 cells was 68 nM and for one of the pre-selected doxorubicin resistant clones was 120 nM. 10-20 nM doxorubicin was employed based on the previously known reports [23,24]. To examine the time-dependent effect of doxorubicin on cell proliferation, ~1000 cells were then incubated with 10 nM doxorubicin for 15 days, and further extended up to 8 weeks. Compared to the doxorubicin-non treated cells, the death rate of doxorubicin-treated cells was gradually reduced up to day 9, and eventually the cell growth rate exceeded the control at day 12 (Figure 1B). However, an abnormally high proliferation was not observed that accounts for a typical aggressive tumor mass. The idea behind the clonal evolution model is that neoplasms continue to divide and fail to die until they are well adapted. Therefore, a shorter doubling time would not necessarily confer an additional survival advantage, especially in the development of acquiring drug resistance. The ratio of MCF-7 cells with the multiple nuclei is significantly increased, before doxorubicin-resistant MCF-7 clones emerged, compared to the drug sensitive control MCF-7 cells. This indicates that certain degree of genomic instability is accompanied by the drug resistance phenotype after a single low dose exposure to cancer cells over the certain period of time. The doxorubicin-induced cell fusion events were further examined using two MCF-7 transfectants having pcDNA3-EGFP/YB-1 or pDsRed2-N1/YB-1.

### Doxorubicin-induced homotypic and heterotypic cell fusion

**Figure 2:**
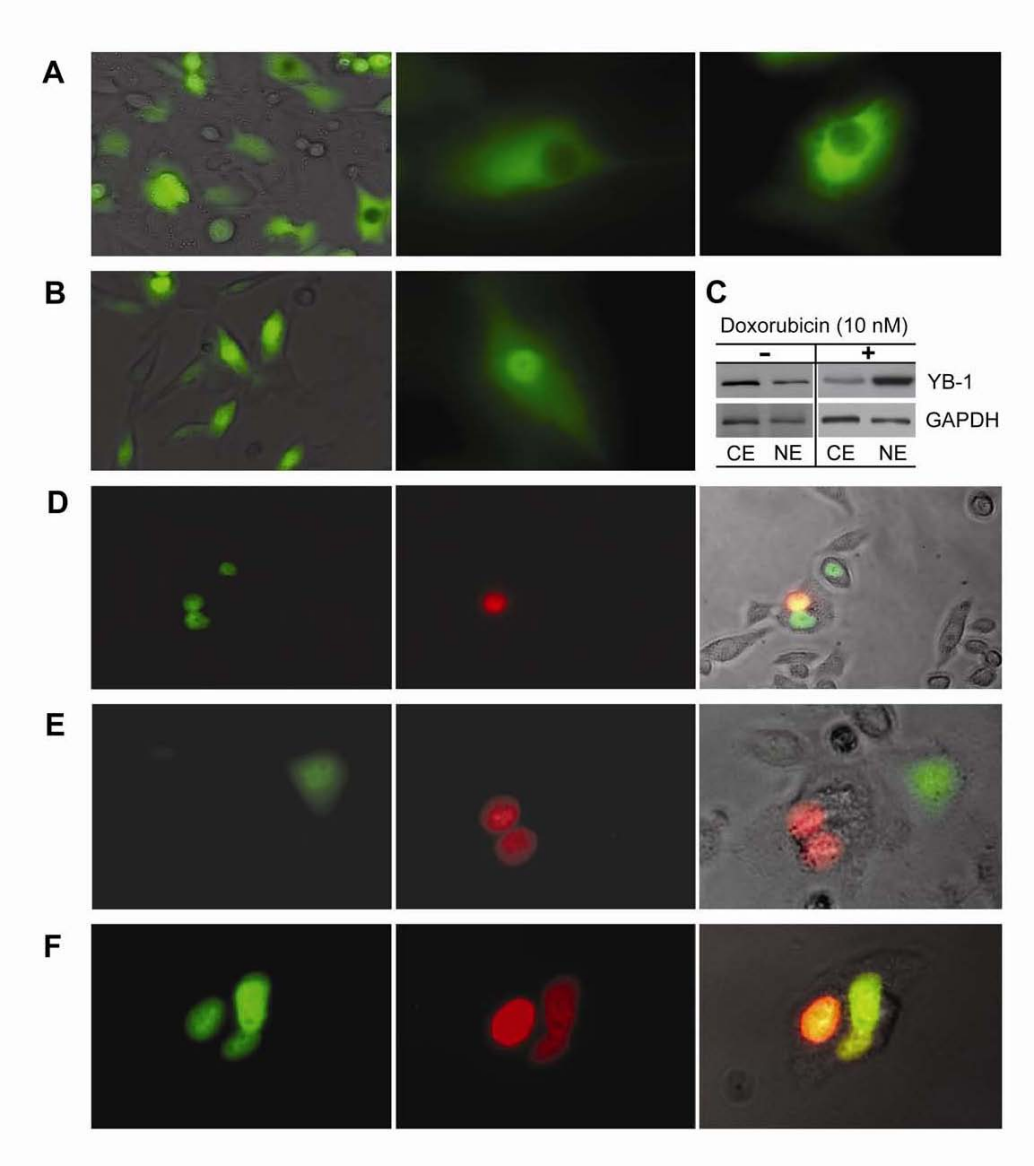
**Doxorubicin-induced cell fusion. Translocation of YB-1-GFP and YB-1-Ds-Red fusion protein in respond to doxorubicin treatment**. Confocal images of the two fluorescent tagged YB-1 proteins localized to each of the nuclei of non-fused MCF-7 cells and localized in one nucleus in fused MCF-7 cells. A: Control; MCF-7/YB-1-GFP transfectant without doxorubicin treatment. B--F: Demonstrate nuclear translocation of YB-1. C: Western blots of YB-1 protein from the fractionated samples; 1 × 10^6 ^cells were lysed using Pierce Nuclear and Cytoplasmic Extraction Reagent Kit. 10 μg of each cytosolic extract; CE, nuclear extract; NE proteins were analyzed by 10% SDS-PAGE and Western blotted using specific antibodies diluted 1:1000 (YB-1) or 1:10000 (GAPDH). Secondary antibody (anti-mouse) diluted 1:25,000 was used with chemiluminescent substrate for detection. Cells were grown for 6 days before visualized by phase contrast and fluorescence microscopy (Axiovert 200, Carl Zeiss). D--F: MCF-7/pcDNA3-EGFP/YB-1 and MCF-7/pDsRed2-N1/YB-1 cells were mixed (1 to 1 ratio) and co-cultured with 10 nM doxorubicin and visualized by phase contrast and fluorescence microscopy. Green; GFP, Red; dsRed, Left panels; GFP detection. Middle panels; dsRed detection. D and F: Right panels; merged (yellow or orange) images represent fused MCF-7 cells.

Two MCF-7 transfectants (transfected either with pcDNA3-EGFP/YB-1 or with pDsRed2-N1/YB-1, Figure 2) were co-cultured (1 to 1 ratio) with or without 10 nM doxorubicin for 6 days and visualized by phase contrast and fluorescence microscopy (Figure 2). In accordance with previous studies [25], YB-1 was expressed almost evenly in cytosol of MCF-7/YB-1 transfectants when there was no drug-related stress (Figure 2A). However, YB-1 localized mostly to the nucleus (Figures 2B and 2C) in the doxorubicin treated cells. After doxorubicin treatment, some cells maybe underwent homotypic cell fusion, resulting in binucleated (DsRed stained) cells in one cytosolic body (Figure 2E) and others further went through nuclear fusion (Figures 2D, E and 2F). However, we cannot exclude that some of these homotypic multinucleated cells may also associate with mitotic defects or with cytokinesis failure. Interestingly, the control MCF-7 cells (cultured in drug free condition) and doxorubicin-resistant MCF-7 cells (still have nuclear expression of YB-1) did not generate fused cells in the same culture condition.

### Heterogeneity of MCF-7 cell populations after acquiring the drug resistance

**Figure 3:**
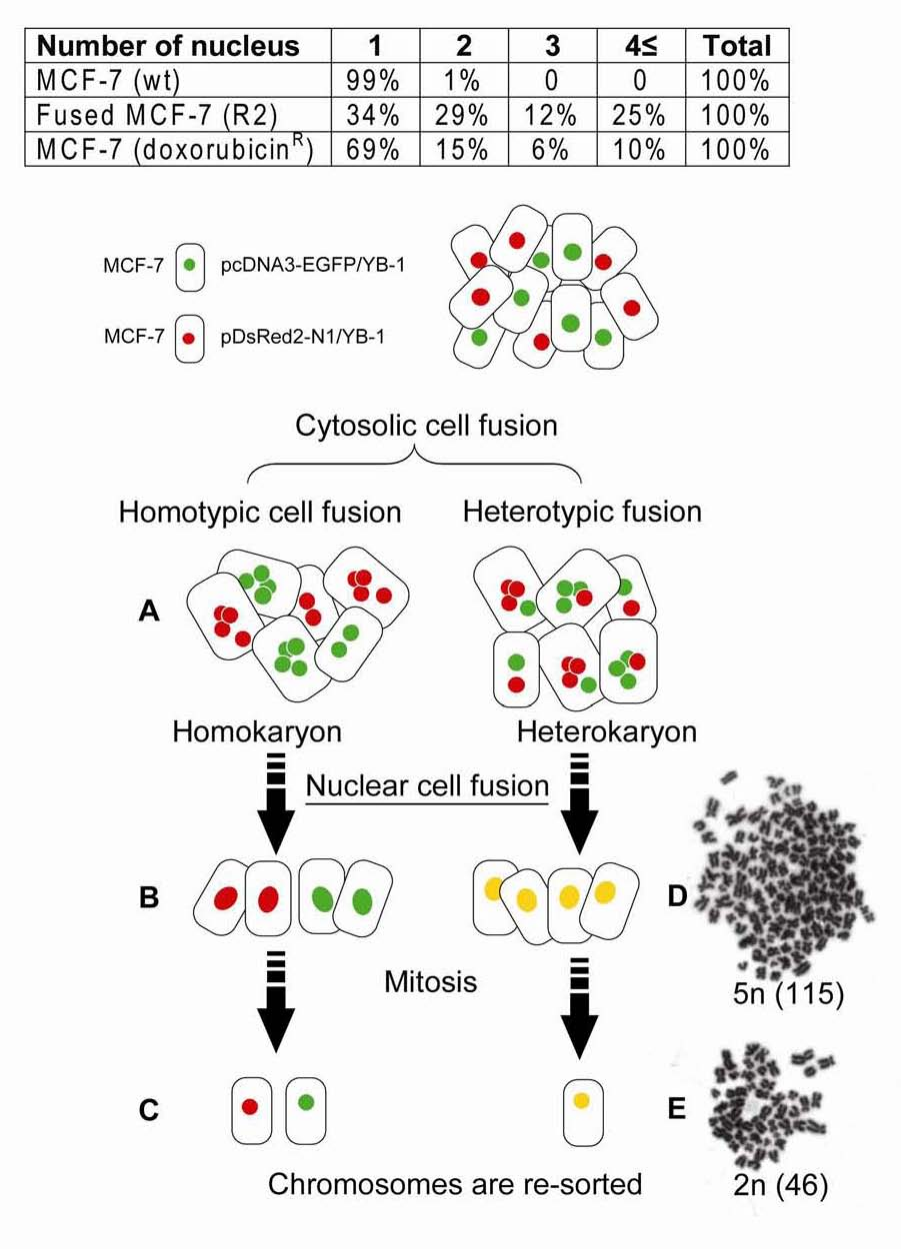
**Schema of cell fusion and chromosomal instability**. A: Cytosolic cell fusion of two subtype of cells (homotype or heterotype). B and D: Most of cells, at the stage of nuclear cell fusion, revealed no further propagation and subsequently vanished. C: Viable progeny cells propagated again in culture. Giemsa-stained chromosomes of MCF-7 cells were examined. D: Abnormal sets of chromosomes as a consequence of cell fusion and (E) the cells containing giant nuclei (polyploid) were died out and the diverse populations were analogously converged into doxorubicin resistant clones. Table insert, fusion was quantitated by counting the number of cells (%) and nuclei present in a microscope field.

**Figure 4:**
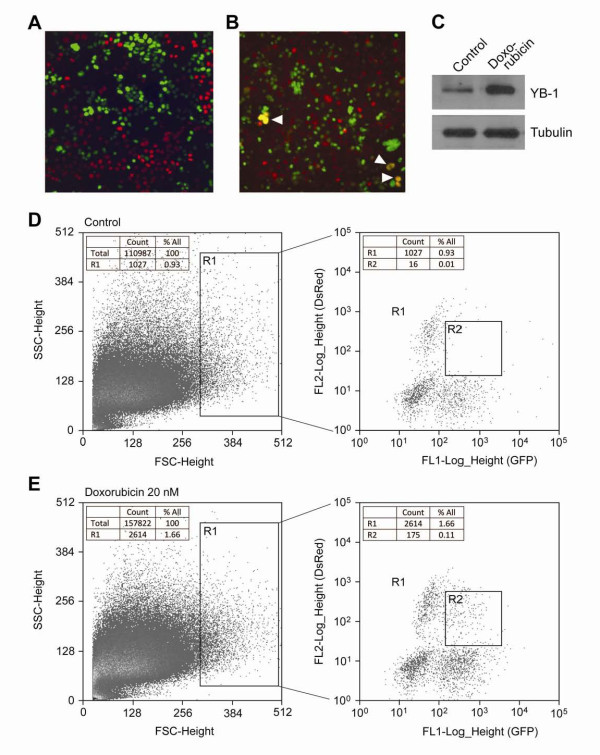
**Cell to cell fusion occurred after doxorubicin treatment**. A: MCF-7/pcDNA3-EGFP/YB-1 and MCF-7/pDsRed2-N1/YB-1 cells were co-cultured (1 to 1 ratio) without doxorubicin, (B) co-cultured with 10 nM doxorubicin. Arrow heads indicate fusion events (merged colour of green and red). C: Doxorubicin induced YB-1 overexpression; each cell lysates from (A and B) were separated by 10% SDS-PAGE, visualized by Western blot using YB-1, tubulin specific antibodies and secondary antibody (anti-mouse) with chemiluminescent substrate. D and E: FACS isolation of fused cells from the co-cultures, gated first (R1) for their size, then the gated merged colored cells (R2) were sorted. GFP fluorescence is plotted on the x-axis (FL1-Log_Height), while red fluorescence is plotted on the y-axis (FL2-Log_Height). The cells (gated R1) represent for the relatively large size of cell population. Double positive cells are gated in R2 region represent hybrids.

**Figure 5:**
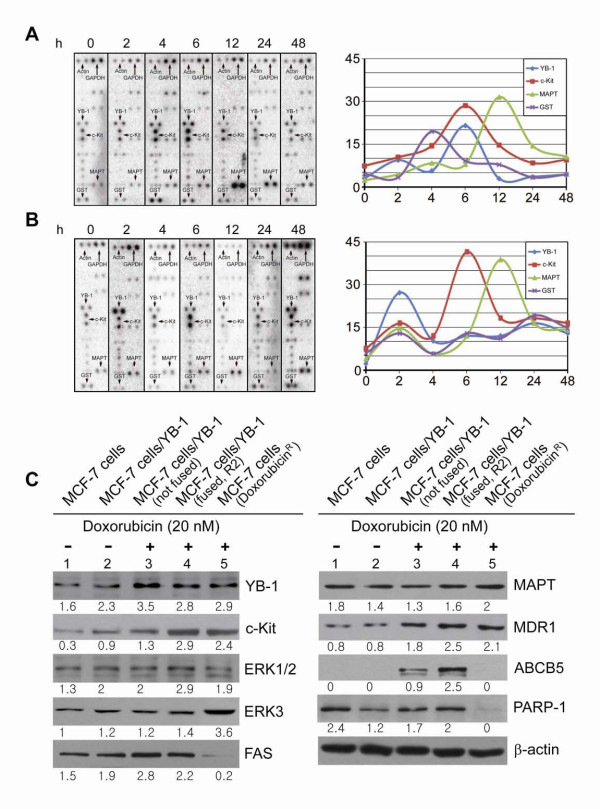
**Differential expression patterns of YB-1, c-Kit, MAPT and GST in time course**. MCF-7 cells that incubated with 20 nM doxorubicin for the indicated periods of time revealed different kinetic patterns for YB-1 and GST expressions between sensitive and resistant MCF-7 cells. A: Dot blot array analysis on doxorubicin sensitive MCF-7 cells. B: Dot blot array analysis on the doxorubicin resistant MCF-7 cells. A and B: The scale on x-axis is not in proportion with time. In the time course study, actin was employed as a control for normalization, because GAPDH was regulated in doxorubicin resistant MCF-7 cells. C: Effect of doxorubicin on the expression of drug resistance related target proteins YB-1, c-Kit, ERK1/2, ERK3, FAS, MAPT, MDR1, ABCB5 and PARP-1 in the four subtypes of MCF-7 cells. 1, 2 are MCF-7 and MCF-7/vector-YB-1 respectively without treatment of doxorubicin. 3, MCF-7/vector-YB-1 with treatment of doxorubicin for 6 h (not fused cells); 4, MCF-7/vector-YB-1 with treatment of doxorubicin for 6 h (fused cells, FACS sorted R2); 5, doxorubicin resistant MCF-7 cell line. Numbers indicate a relative level of protein expression based on the level of intensity of β-actin after normalization.

The appearance of polyploidy cells may be caused by cell-to-cell fusion, we employed flow cytometry to test this assumption and to quantify the cell fusion event occurring after drug treatment. Each MCF-7 subtype, labeled with either EGFP/YB-1 or DsRed/YB-1, were co-cultured as a single monolayer. Figures 3A, B, figure 4A and 4B show that some of these cells were fused and yielded large multicellular syncytia after treatment with 10 nM doxorubicin for 6 days. Fractions of MCF-7 cells containing the multiple nuclei were increased after doxorubicin treatment (inserted table in Figure 3). The polyploidy cells having heterokaryons (Figure 4B) were identified and quantified by FACS sorting (Figures 4D and 4E). In order to increase cell to cell fusion ratio, 20 nM doxorubicin applied (Figure 4E and figure 5C). Initially, 66% out of R2 fraction containing fused cells was polyploidy, however, its ratio was decreased down to 31%, suggesting that chromosomes were eventually lost or re-arranged (Figure 3C and 3E) while acquiring the doxorubicin resistance (Table insert, Figure 3). The results demonstrate cell fusion events, followed by genome re-arrangement. The origin of cancer-initiating cell (cancer stem cell) remains elusive and the fusion of genetic and cytoplasmic material between cells could be important in the development of the cancer stem cell [26].

### Induced expression of cell-fusion associated genes after doxorubicin treatment

Based on the results of Sentrix Human-6-V3 Expression Bead Chips on cell fusion (Additional file 1, Table S1) and literature review, we analyzed the expression patterns of the following genes (YB-1, c-Kit, MAPT, GST, GM-CSF2RA, 15-LOX-1, CypB, LCN2, GRP78, RSK1, COX2, and CRABP2 including controls Actin, GAPDH and guide dots), using custom made dot blot array containing 14 cDNA spots. In the time course study, GAPDH could not be acceptable for normalization because it was regulated in doxorubicin resistant MCF-7 cells. In the pre-chemotherapeutic (doxorubicin sensitive) MCF-7 cells, total 4 genes (YB-1, c-Kit, MAPT, GST) out of 14 were differentially expressed at specific time points after doxorubicin-treatment (Figure 5A). The results were further verified by RT-PCR analysis and the expression patterns of 4 genes, in time course, were similar with the results of dot blot array analysis (data not shown). Genes such as YB-1 and GST revealed transient and inducible kinetics whose level peaked at 6 h and 4 h, respectively in the doxorubicin-sensitive MCF-7 cells. GST was known to detoxify several chemotherapeutic drugs. Therefore, the shift of over-expression of GST, from 4 h to 24 h in the doxorubicin resistant MCF-7 cells (Figure 5B), might be a result of the counter-apoptotic exertion by resisting to oxidative stress-mediated cytotoxicity of doxorubicin [27]. Meanwhile, levels of c-Kit and MAPT were also up-regulated after doxorubicin treatment but with different kinetics from that of YB-1 and GST.

### Expression analysis of drug resistance-related proteins

To investigate whether and how the pro-survival (c-Kit, ERK1/2, ERK3, MAPT, MDR1, ABCB5) and pro-apoptotic genes (FAS and PARP-1) are involved in doxorubicin-induced molecular events (Figure 5C), we examined their expression patterns in each subtype of MCF-7 cells (MCF-7, MCF-7/YB-1, MCF-7/YB-1 not fused, MCF-7/YB-1 fused, and doxorubicin resistant MCF-7 cells). Cells were treated with doxorubicin for 6 h and probed with antibodies against YB-1, c-Kit, ERK1/2, ERK3, FAS, MAPT, MDR1, ABCB5 and PARP-1. YB-1, c-Kit and MDR-1 was noticeably expressed following the drug treatment. However, the expression of FAS and PARP-1 was not detected in the pre-selected doxorubicin resistant MCF-7 cells even after doxorubicin treatment, suggesting that these cells are resistant to drug-induced apoptosis. Of note, ABCB5 expression was induced upon drug treatment in the drug sensitive cells (regardless of non-fused and fused cells), and ABCB5 expression was not induced in the drug untreated sensitive MCF-7 cells and in the pre-selected doxorubicin resistant MCF-7 cells (Figure 5C). Additional efforts are needed to adequately address this disparity. A specific role of ABCB5 in cell fusion, through membrane potential regulation, was reported [22]. If it is the case that the transcription factor YB-1 regulates ABCB5, doxorubicin treatment may correlate with cell fusion, however, further study is needed.

Most of the long term cultured drug resistant cells (fused hybrid, R2), that isolated from the parental MCF-7 cells, were anchorage independent. But some of the anchorage dependent MCF-7 cells exhibited several unique morphological appearances (data not shown). The studies on copy number variation, between non-fused and fused MCF-7 cells, are underway to further define the correlations between the increased genomic instability (by cell fusion) and drug resistance.

## Discussion

Transient neoplastic tumor cells frequently have mass chromosomal alterations, rather than several dozen genetic mutations which eventually lead to drug resistance [13]. During the generation of doxorubicin resistant MCF-7 clones, we observed the presence of multiple nuclei, in the enlarged cytoplasm of cells. The event of nucleus redistribution of the multi-nuclear cells is triggered by cell fusion or by mitotic failure [15] that accounts for the plasticity of adult stem cells *in vivo *[12]. The cell to cell fusion is a newly recognized phenomenon thought to contribute to drug resistance potential. However, molecular mechanisms governing cell fusion are unknown yet. YB-1 was mostly localized to the nucleus rather than to the cytoplasm in the doxorubicin treated doxorubicin-sensitive MCF-7/YB-1 cells. Multi-nucleated cells were generated later through cytosolic cell fusion or some cells further went through nuclear fusion.

Both control and doxorubicin-resistant MCF-7 cells did not noticeably generate fused cells when cultured in drug free condition, suggesting MCF-7 cells with doxorubicin-resistant potential are initiated from the drug induced cell fusion.

YB-1 is frequently expressed in breast cancers [28,29]. When YB-1 is overexpressed in the mammary gland of transgenic mice, breast tumors develop in all of mice [15]. In the initial diagnosis, breast cancers were known to consist of multiple subpopulations of cells with the characteristics of chromosome aberrations [30]. Up to now, there has been no clear explanation on how this diversity comes out and how it is re-arranged in breast cancers. Multi-nucleated cells were generated in doxorubicin treated MCF-7/YB-1 cells (doxorubicin sensitive). When two cells were fused, daughter cells inherited characteristics of heterokaryons or of synkaryons. The heterokaryotic nuclear fusion (0.11%) of two MCF-7 subtypes was identified and quantified by flow cytometry. Figure 4C demonstrates a critical role for YB-1 in MCF-7 cell fusion. Among the doxorubicin treated drug sensitive MCF-7 cells, fused cells (syncytia) have multiple nuclei within a large cytoplasm, and 66% among FACS sorted R2 cells were mainly polyploidy. Some chromosomes were eventually lost or re-arranged in these polyploidy MCF-7 cells, which resulted in the development of doxorubicin resistance. Therefore, drug-resistance might be a severe secondary disease occurring after a series of alterations in chromosome structure and number [9]. Over a century ago, aneuploidy was found in human cancers describing that the abnormal chromosome number is a cause of cancer rather than an end result [31].

Chromosome instability is believed to be responsible for the genome-wide changes of transient stages between early tumorigenesis and subsequent occurrence of aggressive tumor phenotypes [32,33]. Among these small subpopulations of MCF-7 cells, a few cells became resistant to doxorubicin, after an initial response (acquired resistance). However, the reason that some tumor cells are inherently resistant (intrinsic resistance) without previous drug treatment is not known yet. If anti-cancer drug resistant cells represent marginal populations, the present chemotherapeutic treatment should be concentrated on this minor cell population rather than the impending large population of non-drug resistant cells. Further more, spontaneously occurring cell fusion without doxorubicin treatment may suggest a correlation with intrinsic drug resistance.

The overexpression of *P*-Glycoprotein was closely associated with multi-drug resistance [34]. Recently, the amino acid sequence and protein structure of *p-*Glycoprotein ABCB5 was reported [22] and the sequence of ABCB5 is highly homologous (73%) to both of the known human *p-*Glycoprotein isoforms ABCB1 (MDR1) and ABCB4 (MDR3). We were interested in third member of the human *P*-Glycoprotein family ABCB5 (MDR3), because ABCB5 was known to be a determinant of membrane potential and a possible regulator of cell fusion in a defined progenitor subpopulation [22]. Moreover, in the development of drug resistance, the correlation between nuclear YB-1 expression and *P*-Glycoprotein ABCB5 has never been demonstrated. We sought to further confirm the role of ABCB5 in the correlation of pro-survival genes (c-Kit, ERK1/2, ERK3, MAPT and MDR1) with pro-apoptotic gene products (FAS and PARP-1) in YB-1 mediated stimulation by doxorubicin. There have been controversial studies describing fused cells were not tumorigenic [35], until more conclusive data were available describing that hybrids become tumorigenic if they lose certain chromosomes [36]. YB-1 and GST revealed different kinetic patterns on doxorubicin sensitive MCF-7 from those on doxorubicin-resistant MCF-7 cells. On doxorubicin treatment, each peak of the expression levels of YB-1 and GST were shifted to 2 h and 24 h, respectively in the doxorubicin-resistant MCF-7 cells. The turn of YB-1 into earlier response was correlated with nuclear localization of YB-1 that regulates the expression of anti-apoptotic genes. Moreover, the shift of GST expression from 4 h to 24 h was associated with reduced apoptosis, and that may due to detoxification by GST [18]. In this context, survival functions seemed strengthened by the suppressed expression of pro-apoptotic FAS and PARP-1 proteins in doxorubicin resistant MCF-7 clones. Upon drug treatment, YB-1, c-Kit, and MDR1 were all up-regulated compared to doxorubicin non-treated cells. Akt activation was suggested to regulate the nuclear translocation of transcription factor YB-1, affecting the expression of MDR1. A novel finding of our study is the demonstration that c-Kit is co-expressed with ERK3 among drug resistant MCF-7 subpopulations. On the other hand, *P*-Glycoprotein ABCB5 was highly expressed only in the fused MCF-7 cells (R2) while preferentially suppressed in the doxorubicin resistant cells. However, further experiments are necessary to confirm whether ABCB5 is mediated by the expression of YB-1. Ectopically expressed ABCB5 are known to mediate the hyper-polarization of the membrane potential [37]. Other studies illustrated depolarizing the cell line P388 increased the doxorubicin uptake in the sensitive cells but not in the resistant cells [38]. More recently, the association between hyperpolarization of membrane and cell fusion was demonstrated [39]. Taken as a whole, ABCB5 may not directly potentiate doxorubicin resistance, but responsible for increasing heterogeneity in the cancer cell population. In the doxorubicin resistant cells, the intensity of ERK3 band was 2.6-fold increased than that of fused cells. Despite the fact that ERK3 is about 50% identical to ERK1 and ERK2 in its catalytic core [40], key properties of ERK3 is different from the two classical MAPKs. Like YB-1, ERK3 also translocates to the nucleus, upon phosphorylation by MEKs, then ERK3 is constitutively localized to the nucleus [41]. In contrast to ERK1 and ERK 2, the regulation of ERK3 expression is independent of the p53, Bcl-2 and caspase 3, but dependent of p38 pathway activation [40]. Furthermore, the ERK-3 is known to be susceptible to synthetic antagonists.

Our results of current study indicate only a small subpopulation of fused cells obtain acquired resistance, subsequently became stable cell lines having viability against doxorubicin. Therefore, alterations of epigenetic regulation could be another factor that contributes to tumor cell diversity [42].

Through cell fusion, a genome causing drug resistance possibly gets a chance of cellular reprogramming to hide from chemotherapy and re-emerge as a new type of drug resistant cancer after the first targeted cancer cells were eliminated by the therapy. As suggested by other study, the recurred cancers are new hybrid species which might have evolved from their parental cells [43]. The doxorubicin resistant-fused hybrid cells were mostly anchorage-independent and a rare population of the anchorage-dependent MCF-7 cells exhibited differentiated-progenitor cell-like behavior, suggesting that acquired doxorubicin resistant cells may be originated from the fused cells, but additional studies should address this possibility. Due to the complexity of its randomness of acquisition or loss of genetic traits, we can not explain yet how the fate or plasticity of cancer cells is exactly affected by the cell fusion procedure.

## Conclusion

During the generation of doxorubicin resistant MCF-7 clones, multiple nuclei were detected in MCF-7 cells. Exogenously expressed YB-1 was mostly localized to the nucleus rather than to the cytoplasm in the doxorubicin treated doxorubicin-sensitive MCF-7/YB-1 cells. We confirmed the multi-nuclear cells were originated from cell fusion that accompanied by regulation of YB-1, GST, ABCB5 and ERK3. Unlike in ovarian cancer, breast cancer is composed of more diversified subpopulation of cells suggesting stem cell like cells may exist. Cell fusion increased diversity within the MCF-7 cell population and some of the fused cells exhibited progenitor cell like characteristics. Drug resistance could not be explained only by reductions of drug accumulation. The present study shows a correlation between doxorubicin induced transcription factor YB-1 and the transiently regulated fusogenic factor ABCB5 [22], suggesting cell fusion and clonal selection may involve as an additional mechanism in the progress of acquired drug resistance in MCF-7 cells. By understanding the mechanism of drug resistance and discovering novel targets for pharmacological, molecular, and genetic intervention, it may become possible to increase the usefulness of existing anti-cancer drugs.

## Abbreviations

YB-1: Y-box binding protein-1; GST: Glutathione S Transferase; ABCB5: ATP-binding cassette, sub-family B member 5; ERK3: Extracellular signal-regulated kinase 3.

## Competing interests

The authors declare that they have no competing interests.

## Authors' contributions

JYY and JWK designed the research. JYY, SH and YY carried out the collection and assembly of data. JYY, SH, YY and JWK analysed and interpreted the data. JYY and JWK wrote the manuscript. All authors read and approved the final manuscript.

## Pre-publication history

The pre-publication history for this paper can be accessed here:

http://www.biomedcentral.com/1471-2407/10/388/prepub

## Supplementary Material

Additional file 1 **List of regulated genes in the fused MCF-7 cells**. Comprehensive analysis of genome-wide expression on Human Sentrix-6 V3 BeadChip with probes of gene-specific 50 mer oligonucleotides. Based on gene expression ratios (fold x < -2 or x > 1.5), 22 genes (out of 48,803 human genes) were selected as regulated from the fused MCF-7 cells. The numbers in the table represent fold of regulation in comparison with control (non-fused MCF-7). The positive and negative values indicate up- and down-regulation, respectively. Click here for file

## References

[B1] Jasmin C Gil-Delgado MA Marino JP Ecstein E Descorps-Declere A Misset JL Phase I-II constant infusion of adriamycin (doxorubicin) by ambulatory pump delivery system in heavily pretreated (including adriamycin) breast cancer patients Ann Oncol 1990 1 189 193 2261365 10.1093/oxfordjournals.annonc.a057719

[B2] White SC Lorigan P Middleton MR Anderson H Valle J Summers Y Burt PA Arance A Stout R Thatcher N Randomized phase II study of cyclophosphamide, doxorubicin, and vincristine compared with single-agent carboplatin in patients with poor prognosis small cell lung carcinoma Cancer 2001 92 601 608 10.1002/1097-0142(20010801)92:3<601::AID-CNCR1360>3.0.CO;2-K 11505405

[B3] Kruh GD Introduction to resistance to anticancer agents Oncogene 2003 22 7262 7264 10.1038/sj.onc.1206932 14576836

[B4] O'Driscoll L Clynes M Molecular markers of multiple drug resistance in breast cancer Chemotherapy 2006 52 125 129 10.1159/000092540 16612055

[B5] Clarke R Dickson RB Brünner N The process of malignant progression in human breast cancer Ann Oncol 1990 1 401 407 2083184 10.1093/oxfordjournals.annonc.a057790

[B6] Coley HM Mechanisms and consequences of chemotherapy resistance in breast cancer Eur J Cancer 2009 Supplements 7 3 7

[B7] Langley RR Fidler IJ Tumor cell-organ microenvironment interactions in the pathogenesis of cancer metastasis Endocr Rev 2007 28 297 321 10.1210/er.2006-0027 17409287

[B8] Struckhoff AP Bittman R Burow ME Clejan S Elliott S Hammond T Tang Y Beckman BS Novel ceramide analogs as potential chemotherapeutic agents in breast cancer J Pharmacol Exp Ther 2004 309 523 532 10.1124/jpet.103.062760 14742741

[B9] Duesberg P Li R Sachs R Fabarius A Upender MB Hehlmann R Cancer drug resistance: The central role of the karyotype Drug Resist Update 2007 10 51 58 10.1016/j.drup.2007.02.003 17387035

[B10] Jacobsen BM Harrell JC Jedlicka P Borges VF Varella-Garcia M Horwitz KB Spontaneous fusion with, and transformation of mouse stroma by, malignant human breast cancer epithelium Cancer Res 2006 66 8274 8279 10.1158/0008-5472.CAN-06-1456 16912208

[B11] Bjerregaard B Holck S Christensen IJ Larsson LI Syncytin is involved in breast cancer endothelial cell fusions Cell Mol Life Sci 2006 63 1906 1911 10.1007/s00018-006-6201-9 16871371 PMC11136146

[B12] Pochampally RR Neville BT Schwarz EJ Li MM Prockop DJ Rat adult stem cells (marrow stromal cells) engraft and differentiate in chick embryos without evidence of cell fusion Proc Natl Acad Sci USA 2004 101 9282 9285 10.1073/pnas.0401558101 15197249 PMC438968

[B13] Ogle BM Cascalho M Platt JL Biological implications of cell fusion Nat Rev Mol Cell Biol 2005 6 567 575 10.1038/nrm1678 15957005

[B14] Habibi G Leung S Law JH Gelmon K Masoudi H Turbin D Pollak M Nielsen TO Huntsman D Dunn SE Redefining prognostic factors for breast cancer: YB-1 is a stronger predictor of relapse and disease-specific survival than estrogen receptor or HER-2 across all tumor subtypes Breast Cancer Res 2008 10 R86 10.1186/bcr2156 18925950 PMC2614522

[B15] Bergmann S Royer-Pokora B Fietze E Jürchott K Hildebrandt B Trost D Leenders F Claude JC Theuring F Bargou R Dietel M Royer HD YB-1 provokes breast cancer through the induction of chromosomal instability that emerges from mitotic failure and centrosome amplification Cancer Res 2005 65 4078 4087 10.1158/0008-5472.CAN-04-4056 15899797

[B16] Kohno K Sato S Uchiumi T Takano H Kato S Kuwano M Tissue-specific enhancer of the human multidrug-resistance (MDR 1) gene J Biol Chem 1990 265 19690 19696 1978833

[B17] Chen Y Simon SM In situ biochemical demonstration that p-Glycoprotein is a drug efflux pump with broad specificity J Cell Biol 2000 148 863 870 10.1083/jcb.148.5.863 10704438 PMC2174548

[B18] Grant R James W Ironside glutathione S-transferases and cytochrome P450 detoxifying enzyme distribution in human cerebral glioma J Neurooncol 1995 25 1 7 10.1007/BF01054717 8523085

[B19] Bart J Hollema H Groen HJ de Vries EG Hendrikse NH Sleijfer DT Wegman TD Vaalburg W van der Graaf WT The distribution of drug-efflux pumps, P-gp, BCRP, MRP1 and MRP2, in the normal blood-testis barrier and in primary testicular tumours Eur J Cancer 2004 40 2064 2070 10.1016/j.ejca.2004.05.010 15341980

[B20] Bradbury PA Middleton MR DNA repair pathways in drug resistance in melanoma Anticancer Drugs 2004 15 421 426 10.1097/01.cad.0000127665.74096.93 15166615

[B21] Kim IJ Bae YT Kim SJ Kim YK Kim DS Lee JS Determination and prediction of p-glycoprotein and multidrug-resistance-related protein expression in breast cancer with double-phase technetium-99 m sestamibi scintimammography Oncology 2006 70 403 410 10.1159/000098812 17237619

[B22] Frank NY Pendse SS Lapchak PH Margaryan A Shlain D Doeing C Sayegh MH Frank MH Regulation of progenitor cell fusion by ABCB5 P-glycoprotein, a novel human ATP-binding cassette transporter J Biol Chem 2003 278 47156 47165 10.1074/jbc.M308700200 12960149

[B23] Järvinen TA Tanner M Rantanen V Bärlund M Borg A Grénman S Isola J Amplification and deletion of topoisomerase IIα associate with ErbB-2 amplification and affect sensitivity to topoisomerase II inhibitor doxorubicin in breast cancer Am J Pathol 2000 156 839 847 10702400 10.1016/s0002-9440(10)64952-8 PMC1876829

[B24] Hembruff SL Laberge ML Villeneuve DJ Guo B Veitch Z Cecchetto M Parissenti AM Role of drug transporters and drug accumulation in the temporal acquisition of drug resistance BMC Cancer 2008 8 318 10.1186/1471-2407-8-318 18980695 PMC2596802

[B25] Yahata H Kobayashi H Kamura T Amada S Hirakawa T Kohno K Kuwano M Nakano H Increased nuclear localization of transcription factor YB-1 in acquired cisplatin-resistant ovarian cancer J Cancer Res Clin Oncol 2002 128 621 626 10.1007/s00432-002-0386-6 12458343 PMC12164419

[B26] Bjerkvig R Tysnes BB Aboody KS Najbauer J Terzis AJA The origin of the cancer stem cell: current controversies and new insights Nat Rev Cancer 2005 5 899 904 10.1038/nrc1740 16327766

[B27] Müller I Jenner A Bruchelt G Niethammer D Halliwell B Effect of concentration on the cytotoxic mechanism of doxorubicin-apoptosis and oxidative DNA damage Biochem Biophys Res Commun 1997 230 2254 2257 10.1006/bbrc.1996.5898 9016760

[B28] Wu J Lee C Yokom D Disruption of the Y-box binding protein-1 (YB-1) results in suppression of the epidermal growth factor receptor and Her-2 Cancer Res 2006 66 4872 4879 10.1158/0008-5472.CAN-05-3561 16651443

[B29] Stratford AL Habibi G Astanehe A Jiang H Hu K Park E Shadeo A Buys TP Lam W Pugh T Marra M Nielsen TO Klinge U Mertens PR Aparicio S Dunn SE Epidermal growth factor receptor (EGFR) is transcriptionally induced by the Y-box binding protein-1 (YB-1) and can be inhibited with Iressa in basal-like breast cancer providing a potential target for therapy Breast Cancer Res 2007 9 R61 10.1186/bcr1767 17875215 PMC2242657

[B30] Persson K Pandis N Mertens F Borg A Baldetorp B Killander D Isola J Chromosomal aberrations in breast cancer: a comparison between cytogenetics and comparative genomic hybridization Gene Chromosome Canc 1999 25 115 122 10.1002/(SICI)1098-2264(199906)25:2<115::AID-GCC7>3.0.CO;2-2 10337995

[B31] Boveri T Concerning the origin of malignant tumours by Theodor Boveri. Translated and annotated by Henry Harris J Cell Sci 2008 121 Suppl 1 1 84 10.1242/jcs.025742 18089652

[B32] Loeb KR Loeb LA Genetic instability and the mutator phenotype studies in ulcerative colitis Am J Pathol 1999 154 1621 1626 10362784 10.1016/S0002-9440(10)65415-6 PMC1866616

[B33] Gollin SM Chromosomal instability Curr Opin Oncol 2004 16 25 31 10.1097/00001622-200401000-00006 14685089

[B34] Gerlach JH Bell DR Karakousis C Slocum HK Kartner N Rustum YM Ling V Baker RM P-glycoprotein in human sarcoma: evidence for multidrug resistance J Clin Oncol 1987 5 1452 1460 2887642 10.1200/JCO.1987.5.9.1452

[B35] Giguère L Morals R On suppression of tumorigenicity in hybrid and cybrid mouse cells Somat Cell Genet 1981 7 457 471 10.1007/BF01542990 7280931

[B36] Duelli D Lazebnik Y Cell fusion: A hidden enemy? Cancer Cell 2003 3 445 448 10.1016/S1535-6108(03)00114-4 12781362

[B37] Wang L Zhou P Craig RW Lu L Protection from cell death by mcl-1 is mediated by membrane hyperpolarization induced by K(+) channel activation J Membr Biol 1999 172 113 120 10.1007/s002329900589 10556359

[B38] Bose R Lam HYP Membrane transport changes in an adriamycin-resistant murine leukemia cell line and in its sensitive parental cell line Cancer Chemoth Pharm 1988 21 14 18 10.1007/BF00262731 3342461

[B39] Cho HC Kashiwakura Y Marbán E Creation of a biological pacemaker by cell fusion Circ Res 2007 100 1112 1115 10.1161/01.RES.0000265845.04439.78 17395872

[B40] Robinson MJ Xu Be BE Stippec S Cobb MH Different domains of the mitogen-activated protein kinases ERK3 and ERK2 direct subcellular localization and upstream specificity in vivo J Biol Chem 2002 277 5094 5100 10.1074/jbc.M110935200 11741894

[B41] Khokhlatchev AV Canagarajah B Wilsbacher J Robinson M Atkinson M Goldsmith E Cobb MH Phosphorylation of the MAP Kinase ERK2 promotes its homodimerization and nuclear translocation Cell 1998 93 605 615 10.1016/S0092-8674(00)81189-7 9604935

[B42] Kikyo N Wolffe AP Reprogramming nuclei: insights from cloning, nuclear transfer and heterokaryons J Cell Sci 2000 113 11 20 10591621 10.1242/jcs.113.1.11

[B43] Pawelek JM Tumor cell fusion as a source of myeloid traits in cancer Lancet Oncol 2005 6 988 993 10.1016/S1470-2045(05)70466-6 16321767

