# Supplementary figures and images for: p-Glycoprotein ABCB5 and YB-1 expression plays a role in increased heterogeneity of breast cancer cells: correlations with cell fusion and doxorubicin resistance
Source: BMC Cancer. 2010 Jul 22;10:388. doi: 10.1186/1471-2407-10-388 (PMC2913965; doi:10.1186/1471-2407-10-388)

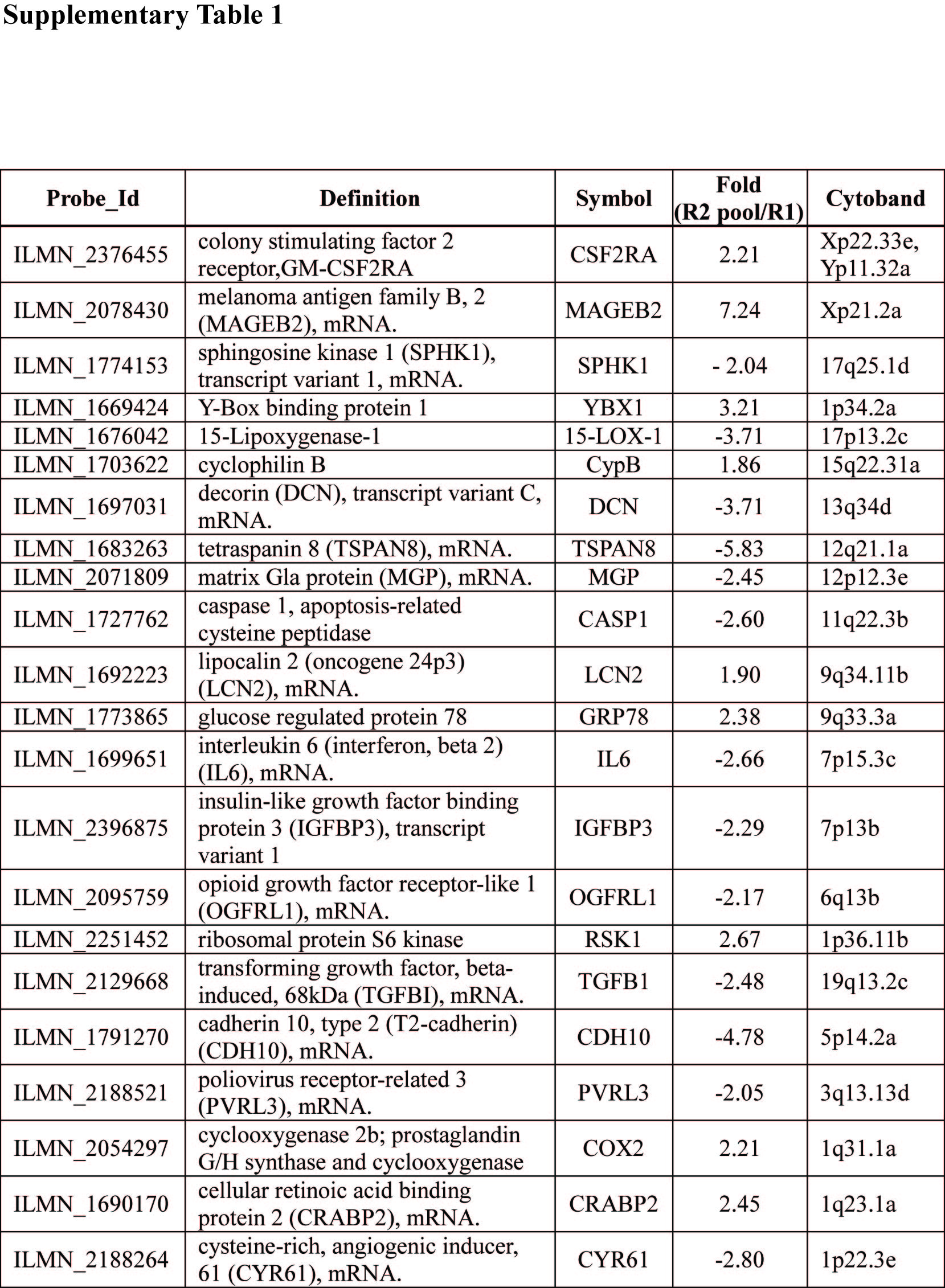

Supplement: Additional file 1 — List of regulated genes in the fused MCF-7 cells. Comprehensive analysis of genome-wide expression on Human Sentrix-6 V3 BeadChip with probes of gene-specific 50 mer oligonucleotides. Based on gene expression ratios (fold x < -2 or x > 1.5), 22 genes (out of 48,803 human genes) were selected as regulated from the fused MCF-7 cells. The numbers in the table represent fold of regulation in comparison with control (non-fused MCF-7). The positive and negative values indicate up- and down-regulation, respectively. [file 1471-2407-10-388-S1.JPEG]
